# The Six-Facet Artificial Intelligence Literacy Questionnaire (SFAILQ): Assessing AI Literacy in Adolescents, Young Adults, and Midlife Adults

**DOI:** 10.3390/bs16071110

**Published:** 2026-07-03

**Authors:** Qingqi Liu, Wenjiao Miao, Jingjing Li, Yuju Lei

**Affiliations:** 1 Bay Area School of Applied Psychological Sciences, Beijing Normal University at Zhuhai, Zhuhai 519087, China; liuqingqi@bnu.edu.cn (Q.L.); 202521061130@mail.bnu.edu.cn (W.M.); 2 Faculty of Psychology, Beijing Normal University, Beijing 100875, China; 3 College of Education for the Future, Beijing Normal University at Zhuhai, Zhuhai 519087, China; 202238039072@mail.bnu.edu.cn; 4 School of Cultural Communication and Design, Guangdong Open University, Guangzhou 510091, China; 5 School of Teacher Education, Hubei University of Arts and Science, Xiangyang 441053, China

**Keywords:** AI literacy questionnaire, affective experiences, usage skills, cognitive evaluation, ethical norms, responsible use, self-development, academic self-efficacy, academic engagement

## Abstract

AI literacy has become a pressing concern across disciplines, calling for comprehensive measurement tools applicable to diverse age groups. Building on existing research, we propose a six-facet model encompassing affective experiences, usage skills, cognitive evaluation, ethical norms, responsible use, and self-development. The present study aimed to develop and validate the Six-Facet Artificial Intelligence Literacy Questionnaire (SFAILQ) among 2443 Chinese participants aged 12 to 60 years, spanning adolescence to middle adulthood, with a disproportionately larger proportion falling within the 18-to-40 age range. An item reduction analysis was conducted using the first split-half sample (*N*_1_ = 1217), and reliability and validity analyses were performed with the second split-half sample (*N*_2_ = 1226). The final SFAILQ consists of 32 items assessing six dimensions: affective experiences (5 items), usage skills (5 items), cognitive evaluation (6 items), ethical norms (6 items), responsible use (4 items), and self-development (6 items). All six dimensions and the total score correlated significantly and positively with academic self-efficacy (usage skills showing the strongest correlation) and with academic engagement (responsible use demonstrating the highest correlation). The SFAILQ demonstrated high internal consistency, construct validity, convergent validity, discriminant validity, and criterion-related validity. It may serve as an effective tool for evaluating AI literacy among adolescents, young adults, and midlife adults.

## 1. Introduction

Despite being in its early stages, the adoption of artificial intelligence (AI) is progressively expanding across numerous domains. AI has gained increasing importance in various sectors, including education and employment (Laupichler et al., 2022; Pereira et al., 2023), daily activities and problem-solving (Kong et al., 2021; Joksimovic et al., 2023; Ramya & Khandelwal, 2024), smartphones and smart homes (Choudrie et al., 2023; Guo et al., 2019), autonomous vehicles, and intelligent healthcare systems (Garikapati & Shetiya, 2024; Yang et al., 2022). A recent survey conducted by McKinsey (2024) revealed that, as of May 2024, more than two-thirds (72%) of respondents in the United States state that their organizations are utilizing AI. By June 2024, the user base of generative AI products in China had expanded to 230 million individuals (China Internet Network Information Center, 2024). Moreover, the global generative AI market size is forecasted to grow from USD 20.9 billion in 2024 to USD 136.7 billion by 2030, at a compound annual growth rate (CAGR) of 36.7% during the forecast period (MarketsandMarkets, 2024). In the context of unprecedented opportunities and challenges, it is crucial to approach AI with an open mind and a constructive attitude, with a focus on learning from and effectively leveraging its capabilities.

### 1.1. What We Know About AI Literacy

AI literacy encompasses an individual’s theoretical knowledge and practical skills pertaining to AI (Zhou et al., 2024). A higher level of AI literacy equips individuals with the ability to effectively leverage AI for both personal and societal development. Within the realm of computer science, AI literacy is focused on an understanding of the underlying concepts and technologies that underpin AI products and services. Conversely, in the field of education, AI literacy is targeted towards the general population, emphasizing individuals’ awareness, skills, attitudes, and ethical considerations surrounding the use of AI products (Cai et al., 2024; Laupichler et al., 2023a; Laupichler et al., 2023b). Long and Magerko (2020) defined AI literacy across five dimensions: “What is AI? What can AI do? How does AI work? How should AI be used? and How do people perceive AI?” They identified 17 core competencies that empower individuals to assess AI technologies critically, communicate and collaborate effectively with AI, and leverage AI tools within various contexts, including online platforms, households, and workplaces. Long and Magerko’s study is one of the first to provide a detailed account of the specific components of AI literacy. Building upon a comprehensive review of the literature on AI literacy, Ng et al. (2021) identified four key aspects: “knowing and understanding AI,” “using and applying AI,” “evaluating and creating AI,” and “AI ethics.” In a subsequent development, Ng et al. (2024) proposed the ABCE model of AI literacy, which comprises four core elements: affective learning, behavioural learning, cognitive learning, and ethical learning. From a distinct psychological perspective, Ng et al. (2021) and Ng et al. (2024) offered a particularly holistic framework for understanding AI literacy, emphasizing the multifaceted psychological dimensions of emotion, behaviour, and cognition while giving significant attention to ethical considerations in the use of AI. In addition to the ABCE model of AI literacy, the KSAVE model, which includes knowledge, skills, attitudes, values, and ethics, has also been emphasized in some studies as a framework for defining AI literacy (Cai et al., 2024; B. Wang et al., 2023). Although a unified consensus on the conceptual framework of AI literacy has yet to be reached, researchers are actively expanding its scope by incorporating more critical factors. From the perspective of the essential components of AI literacy, an integrated definition, drawing upon the contributions of previous researchers, can be articulated as follows: AI literacy is characterized by the presence of positive affective experiences, advanced utilization skills, and well-defined cognitive concepts during the application of AI, all while adhering to ethical standards. As AI technology progresses and becomes more intricately intertwined with daily life, the conceptual framework of AI literacy is poised to evolve further.

Some researchers have developed measurement tools for AI literacy to explore the concept more deeply. Laupichler et al. (2023b) initially employed an iterative Delphi expert method to create a 47-item scale specifically designed to assess AI literacy, with the aim of evaluating individuals’ understanding of AI. However, they did not conduct item discrimination analysis, factor analysis, or reliability analysis, so the validity and reliability of the scale were unverified. Subsequently, Laupichler et al. (2023a) developed an AI literacy test questionnaire for adolescents and adults that encompasses three dimensions: “Technical Understanding,” “Critical Appraisal,” and “Practical Application.” While this latter scale underwent validation and reliability testing, a notable limitation is its omission of AI ethics, a component that many researchers deem essential for a comprehensive measurement. B. Wang et al. (2023), leveraging the KSAVE model and the technological-cognitive-ethical framework, employed rigorous statistical methods to design and create an artificial intelligence literacy scale for both adolescent and adult populations. The scale consists of 12 items that assess four dimensions: “awareness,” “usage,” “evaluation,” and “ethics.” Due to the methodological rigor underlying its development, the scale shows good reliability and validity. However, the primary aim of developing the artificial intelligence literacy scale was to evaluate individuals’ AI literacy in the context of future human–computer interaction (HCI) research. HCI studies often examine AI usage from a programming perspective rather than from a user competency angle (Amershi et al., 2019). As a result, the scale’s validity outside the HCI domain may be questionable (Laupichler et al., 2023a). Using the affective-behavioural-cognitive-ethical (ABCE) model, Ng et al. (2024) developed a four-dimensional AI literacy questionnaire for adolescents aged 12 to 17 years. The scale focuses specifically on AI learning, structuring its four dimensions: affective learning, behavioural learning, cognitive learning, and ethical learning. While literacy has traditionally been defined as the ability to understand and communicate through written language, confining the concept of AI literacy solely within the realm of AI learning may narrow the broader interpretation of AI literacy. Zhou et al. (2024) proposed a measurable description of AI literacy from five dimensions of the KSAVE model (i.e., knowledge, skills, attitudes, values, and ethics) and developed an AI literacy competency measurement scale among university students. However, the scale did not fully align with the five-factor model of the KSAVE assessment framework, as it identified only four factors through empirical exploration. Additionally, the lack of structural validity testing for the scale leaves ambiguity regarding the full support for the four-factor model. Upon summarizing the current measurement tools for AI literacy, researchers have attempted to assess AI literacy from multiple perspectives, developing various versions of AI literacy scales. Undoubtedly, previous endeavours have significantly contributed to advancing quantitative research on AI literacy. However, three critical issues related to AI literacy remain among the current measurement tools that we need to know.

### 1.2. What We Need to Know About AI Literacy

First, no scale that measures responsible use as a core factor currently exists. The powerful capabilities of AI technology require individuals to adopt a responsible approach when using AI products, rather than relying entirely on AI to complete tasks for them. The advanced functionalities of AI have led to its pervasive integration into daily life, surpassing nearly all other technological products. Given its remarkable capabilities, AI has permeated almost every aspect of modern living. Under such circumstances, individuals who refrain from using AI products altogether cannot be considered highly AI-literate. Conversely, excessive reliance on AI—to the point of delegating tasks that demand one’s own careful attention and responsibility—also does not reflect true AI literacy. A critical aspect of AI literacy is that proficient users typically maintain a balanced approach to AI usage, ensuring that it does not excessively disrupt their daily lives while potentially yielding positive outcomes (Chetty, 2023). However, excessive reliance on AI can lead to dependency or even addiction, adversely affecting one’s learning and professional responsibilities (Parsakia, 2023), thereby resulting in irresponsible behaviour. Individuals with high levels of AI literacy generally adhere to a responsible usage model. Recently, the concept of responsible use has garnered increasing scholarly attention in the fields of social media and AI (e.g., Ciriello et al., 2024; Dignum, 2019; Lyons et al., 2023). Some researchers have argued that responsible use encompasses moderate or rational engagement with technology or media. A review of digital literacy suggests that its definition should reflect reasonable usage, which is primarily evident in the intensity of engagement (W. J. Wang et al., 2020). As technological capabilities evolve and their integration into everyday life deepens, the boundaries of what constitutes moderate or reasonable usage may become increasingly blurred. Thus, in addition to considering moderate use, embracing responsibility within the framework of responsible use is crucial, particularly with respect to one’s accountability to oneself and others. Given that discussions surrounding accountability to others frequently encompass ethical considerations, we prioritize the concept of individual responsibility when examining the responsible use of AI. Self-accountability in the context of AI usage entails not transferring one’s obligations, such as task completion, entirely to the AI. Thus, we define responsible use through the lenses of reasonable engagement and self-accountability, characterizing the responsible use of AI literacy as the rational application of AI with a commitment to self-responsibility, thereby avoiding excessive dependence that could disrupt normal life. Our focus on prudent engagement and self-accountability in the responsible use of artificial intelligence aligns closely with the principles outlined in the European Framework for the Digital Competence of Educators (DigCompEdu) (Redecker, 2017), particularly within the core area of “Facilitating Learners’ Digital Competence.” DigCompEdu emphasizes two critical aspects of responsible use: first, ensuring that technology does not adversely affect physical, psychological, or social well-being; and second, utilizing digital technology safely and responsibly. The first aspect underscores the importance of moderating technology use, as excessive engagement can lead to detrimental outcomes, which directly corresponds to our concept of prudent engagement. The second aspect, concerning the safe and responsible use of digital technology, is reflected in our emphasis on self-accountability, which entails avoiding irresponsible AI usage and refraining from delegating responsibilities that should be managed personally to AI products.

Second, no scale for measuring self-development as a fundamental aspect of AI literacy is currently available. The rapid advancement of AI technology necessitates continuous and dynamic development of AI literacy among individuals, particularly by integrating AI literacy with their personal growth in an evolving manner. AI technology is developing at an unprecedented pace, and this trend is expected to persist in the foreseeable future. Such rapid progress requires individuals to stay informed about AI developments and consistently update their knowledge. In other words, the measurement of AI literacy should not only focus on existing competencies (static) but also consider future potential (dynamic). Furthermore, the combination of static abilities and dynamic potential should not be understood merely at an abstract conceptual level—rather, it must be reflected in individual development. The significance of literacy lies in its substantial contribution to personal growth. Directly linking AI literacy with self-development exemplifies the effective integration of theoretical knowledge and practical application. Previous studies on AI literacy have focused primarily on existing knowledge, skills, and ethical considerations (Ng et al., 2021; Zhou et al., 2024), which reflect relatively static levels of ability. However, they have largely overlooked the dynamic nature of the developmental process, particularly concerning self-development. An increasing number of researchers argue that self-development should be a central component of both internet literacy and digital literacy (Berezhna et al., 2021; Biggins et al., 2017; Ma et al., 2024; W. J. Wang et al., 2020). One of the six core domains of the European Framework for the Digital Competence of Educators (DigCompEdu) (Redecker, 2017), “Professional Engagement,” explicitly underscores the importance of “Digital Continuous Professional Development,” which involves utilizing digital sources and resources for ongoing professional growth. The principles of continuity, professionalism, and development highlighted in “Digital Continuous Professional Development” are central to our focus on promoting AI literacy. In AI literacy, self-development involves continually improving AI knowledge/skills and applying AI to daily problem-solving, ultimately enhancing personal development. By prioritizing self-development as a fundamental component, AI literacy can encompass both present competencies and future potential, incorporating both theoretical mastery of AI concepts and practical applications of AI technology.

Third, very few scales have simultaneously focused on adolescents, young adults, and midlife adults. Currently, the main existing AI literacy scales primarily target young and midlife adults (B. Wang et al., 2023; Laupichler et al., 2023a; Laupichler et al., 2023b; Zhou et al., 2024). Only one scale specifically targets adolescents under 18 years old (Ng et al., 2024). To our knowledge, no scale has been developed specifically for concurrently measuring AI literacy among adolescents, young adults, and midlife adults. Although AI literacy scales initially developed for adult populations can be adapted for use in adolescent populations with minor revisions, there is a concern that scales designed for adults may not adequately capture or verify the psychometric characteristics of adolescents. Specifically, when adult scales are revised to suit adolescents, some inappropriate items may need to be removed. The removal of these items may decrease the scale’s ability to measure a greater number of underlying factors effectively. Similarly, AI literacy scales developed for adolescents may encounter analogous issues when applied to adult populations. Only by employing rigorous scale development procedures from the outset, along with conducting item generation and reliability and validity testing in both adolescent and adult populations, can we effectively measure the AI literacy levels of both age groups. The development of AI measurement tools that are applicable to adolescents, young adults, and midlife adults simultaneously could provide future researchers with instruments to analyse and compare differences in AI literacy effectively across different developmental stages, thereby fostering a deeper understanding of AI literacy.

### 1.3. A Novel Framework: The Six-Facet Model

By building upon previous research on AI literacy, particularly the ABCE model, the KSAVE model, and relevant scales, we aim to enhance its fundamental components by integrating two critical factors: responsible use and self-development. We developed the scale items on the basis of a six-facet model including six core factors: affective experiences, usage skills, cognitive evaluation, ethical norms, responsible use, and self-development. Affective experiences refer to the emotional responses tied to AI usage, where greater AI literacy is suggested by more frequent positive emotions and fewer negative emotions, since these directly influence one’s AI-related cognitive beliefs and practical skills. Usage skills denote both subjective confidence and objective competence in operating AI products. Cognitive evaluation represents the interpretative understanding and analytical assessment of AI technology, specifically regarding distinctions between AI/non-AI technologies, AI’s advantages/disadvantages, and usage scenario evaluations. Ethical norms involve adhering to ethical standards and legal regulations to prevent potential risks/harms. Responsible use means employing AI considerately and accountably to avoid negative physical, psychological, and social impacts during digital technology use. Self-development signifies continually updating AI-related theoretical knowledge and practical skills to solve real problems, thus advancing personal growth. Drawing upon the six-facet model, we define AI literacy as the responsible use of AI within ethical standards, characterized by the experience of positive emotions, proficiency in employing AI tools, clear and rational cognitive frameworks, and a commitment to continually enhancing one’s theoretical understanding and practical proficiency in AI for the purpose of fostering self-development. Table 1 systematically juxtaposes the key characteristics of the two main prominent AI literacy frameworks (i.e., the ABCE model and the KSAVE model) and our newly proposed six-facet model. The distinctions between our six-facet model and the ABCE and KSAVE models from prior research primarily encompass two aspects.

First, we incorporated two important factors that have gained increasing emphasis from researchers in recent years but are not specifically addressed by current AI literacy scales: responsible use and self-development. The ABCE model and the KSAVE model primarily define and measure AI literacy across four dimensions: affective, behavioural, cognitive, and ethical. Although the KSAVE model proposes five key factors including knowledge, skills, attitudes, values, and ethics, the two scales derived from that framework (B. Wang et al., 2023; Zhou et al., 2024) do not explore or validate a five-factor structure. B. Wang et al. (2023) identified only four factors, namely “awareness,” “usage,” “evaluation,” and “ethics,” through exploratory and confirmatory factor analyses. Zhou et al. (2024) similarly developed a four-factor model following exploratory factor analysis, comprising AI knowledge, AI skills, AI ethics, and a combined factor of AI attitudes and values, thereby merging the originally distinct dimensions of attitudes and values into one. In our six-facet model, affective experiences, usage skills, cognitive evaluation, and ethical norms align respectively with the affective, behavioral, cognitive, and ethical dimensions of AI literacy as outlined in prior theoretical models. The newly introduced factors of responsible use and self-development enrich the conceptual framework of AI literacy, ensuring its relevance amidst the ongoing evolution of AI technology and its increasing integration into daily life.

Second, compared with the ABCE model, which has been validated primarily within adolescent populations, and the KSAVE model, which is used primarily with adult groups, we aim to extend the applicability of the Six-Facet Model to encompass a broader age range, spanning adolescents aged 12–17, young adults aged 18–40, and middle-aged individuals aged 41–60. Currently, while scales exist that separately measure AI literacy for adolescents and adults, these scales utilize measurement tools grounded in different theoretical models. Consequently, the results obtained from these distinct measurement tools cannot be directly compared, impeding our ability to summarize cross-sectional differences in AI literacy between adolescents and adults. Given the broad age range we aim to cover, we must achieve cross-age equivalence in scales. Nevertheless, a single scale that is applicable across multiple age groups can be developed if careful attention is given to the characteristics of different age demographics during the initial design of scale items, ensuring their suitability for various age stages from the outset. If we can harness our six-facet model concurrently across adolescents, young adults, and middle-aged populations to develop a scale that meets psychometric requirements, it will not only furnish a theoretical framework for future research on AI literacy across multiple age stages but also provide reliable and valid measurement tools. The results derived from this unified measurement tool will facilitate direct comparisons, revealing overall levels of AI literacy and differences within its various sub-dimensions across different age groups. This, in turn, reflects, to some extent, the influence of age-related or temporal changes and societal development on AI literacy.

Beyond the specific context of AI literacy, our six-facet model aligns closely with broader, well-established digital competence frameworks such as the Digital Competence Framework for Citizens (DigComp 2.2) and the Digital Competence Framework for Educators (DigCompEdu). In particular, the dimensions of our model map more directly onto those of DigCompEdu. For example, multiple dimensions in our six-facet model correspond to some extent with the core components of the DigCompEdu, although not in a perfect one-to-one mapping. The affective experiences dimension in our six-facet model and Empowering Learners in DigCompEdu both emphasize focusing on learners’ emotions and motivations to promote autonomous learning of digital technologies; our usage skills dimension and Teaching and Learning in DigCompEdu both stress the core competencies in applying digital technologies; our cognitive evaluation dimension and Assessment in DigCompEdu both emphasize comprehensive understanding and in-depth evaluation of technologies; our ethical norms dimension and Digital resources in DigCompEdu (particularly its components of managing, protecting, and sharing) both highlight adherence to ethical standards; our responsible use dimension emphasizes the reasonable use of AI with a self-accountable attitude, which directly corresponds to Responsible use under Facilitating Learners’ Digital Competence in DigCompEdu; our self-development dimension and Professional Engagement in DigCompEdu (especially its Digital Continuous Professional Development component) both emphasize the continuous improvement of professional capabilities (particularly professional practice skills) through digital technologies. Thus, our six-facet AI literacy theoretical framework largely corresponds with the currently influential digital competence framework worldwide, although their core dimensions are not entirely identical, possibly because the latter focuses on educators and addresses broader digital technologies. Additionally, DigComp 2.2 includes a dedicated section on AI, which emphasizes the importance of understanding fundamental AI principles and developing sensitivity to data protection, privacy, ethics, and bias.

It should be noted that further distinction is needed between the two dimensions, both rooted in the DigCompEdu framework: ethical norms and responsible use. Ethical norms refers to individuals’ internalized understanding and acceptance of AI-related ethical principles, such as fairness, transparency, accountability, and privacy protection. It captures the cognitive-moral dimension of AI literacy—that is, “knowing what is right.” Responsible use, by contrast, refers to the behavioural translation of these ethical understandings into concrete actions when interacting with AI systems. It comprises two interrelated components: prudent engagement, which entails moderate and balanced use of AI without excessive dependence that disrupts normal life and well-being; and self-accountability, which entails not delegating one’s own obligations and responsibilities to AI products. In essence, responsible use addresses the question of “how to act on what is right” in everyday AI practices. The distinction also has a theoretical foundation in DigCompEdu. While ethical norms align with the value-based and principled aspects of digital competence, responsible use corresponds more directly to the practical and behavioural facets emphasized in DigCompEdu, such as safe, responsible, and moderate technology use that safeguards physical, psychological, and social well-being, and avoids transferring personal responsibilities to technology. Thus, although the two dimensions may correlate empirically, they are not identical: ethical awareness does not guarantee ethical behaviour, and distinguishing them allows the SFAILQ to capture both the cognitive-moral and the behavioural-implementation facets of AI literacy.

These six elements correspond well to the cognitive evaluation and ethical norms dimensions of our model, respectively. Mapping the core components of our six-facet theoretical framework onto the well-established model can help us more clearly define each dimension and ensure their consistency with established evaluation standards, thereby enhancing their validity and applicability.

### 1.4. The Present Study

AI literacy has increasingly garnered the attention of researchers, especially within the fields of education and psychology. However, in contrast to other prominent research themes emerging from the mobile internet era, such as problematic mobile phone use and digital literacy, there remains a notable lack of measurement tools for AI literacy. The diverse range of instruments available for assessing problematic mobile phone use and digital literacy has undoubtedly facilitated extensive theoretical and applied research in these areas. In our research, we aim to advance the study of AI literacy by developing a reliable and valid measurement tool, thereby contributing to both theoretical exploration and practical applications in the realm of AI usage. Building upon existing definitions, theoretical models, and measurement tools related to AI literacy, we propose a six-facet model and intend to create an accompanying measurement tool with robust reliability and validity. Furthermore, we seek to investigate and validate the structure and dimensions of AI literacy across a broader age spectrum, ultimately striving to establish a reliable and valid AI literacy scale for adolescents, young adults, and midlife adults. It should be noted that, since adults aged 18–40 are currently the predominant user group of AI, our sample will include a relatively larger proportion of the 18–40 age cohort, while still covering the broader age range from 12 to 60 years.

## 2. Materials and Methods

The present study was performed in line with the principles of the Declaration of Helsinki. Approval was granted by the Ethics Committee of Faculty of Psychology, Beijing Normal University (protocol code BNU202406190121, approval date: 19 June 2024). Informed consent was obtained from all individual participants involved in the study between June and August 2024. For participants under the age of 18, consent was also acquired from their guardians. We developed the AI literacy scale by following the methodological steps outlined by prior researchers (e.g., Hinkin, 1998; B. Wang et al., 2023). These steps included item generation, content validation, questionnaire administration, item reduction, confirmatory factor analysis, and assessments of reliability and validity.

### 2.1. Item Generation

We employed two primary methods for the initial compilation of questionnaire items. First, we reviewed existing AI literacy assessment tools and select and adapt appropriate items for our specific needs. Importantly, we translated the content of the English scales into Chinese using a back-translation process. We also ensured that the scale items are applicable to adolescents, young adults and midlife adults alike; for example, phrases that exclusively address AI use in the workplace would not be suitable for younger cohorts, prompting us to revise terminology to “in study or work.” Second, we independently developed measurement items that target the core factors of AI literacy. During the self-compilation process, our descriptions closely aligned with the conceptual definitions of these core factors. For example, within the dimension of “self-development,” we emphasized both the theoretical knowledge and practical skills necessary for AI-related self-improvement. Additionally, we carefully regulated the number of self-compiled items to maintain an approximately equal initial item count across each dimension. Ultimately, we acquired a total of 70 items.

### 2.2. Content Validation

We recruited four subject matter experts (SMEs) for the content validation analysis. Among them, two hold doctoral degrees in psychology, while the other two possess master’s degrees in psychology and education. While AI literacy is primarily regarded as an educational concept, it is increasingly gaining attention within the field of psychology, where psychology researchers possess a relatively comprehensive understanding of it. Among the four SMEs, three are psychology researchers, and only one is an educational researcher. However, all of them engaged in thorough study and discussion of the concept of AI literacy prior to evaluating the items and reached a consensus on the various dimensions of AI literacy that our study aimed to measure. Thus, the influence of the backgrounds of the four SMEs participating in the item generation phase may be constrained. Furthermore, compared with educational researchers, psychology researchers may have a more profound understanding of the psychometric characteristics of the scales, which can help ensure that the developed scale possesses favourable psychometric properties. A conservative retention criterion was adopted during the initial stage: an item was retained only if all four experts rated it as quite or highly relevant (I-CVI = 1.00) to the intended dimension. The stringent criterion was chosen to ensure that only items with unambiguous relevance and clarity were retained in the initial pool, thereby minimizing content-irrelevant variance at the early stage of scale development (Lynn, 1986). Items that received any rating below “quite relevant” were either revised and re-evaluated by the experts or, in cases where revision was insufficient, removed. Ultimately, all retained items achieved I-CVI = 1.00. The scale-level content validity index (S-CVI/Ave) was 1.00, indicating sound content validity. After the content-validation stage, the experts meticulously examined and discussed the clarity and appropriateness of the wording and format of each item to ensure that the descriptions were accessible to both adults and adolescents. Ultimately, 64 items were revised and finalized to constitute the foundational pool for the AI literacy questionnaire (see Appendix A).

### 2.3. Questionnaire Administration

To include adolescents in our study, we employed convenience sampling to select three high schools. To mitigate the inherent biases associated with convenience sampling, we specifically targeted schools located across three distinct cities, each representing varying levels of economic development according to China’s classification system: first-tier, second-tier, and third-tier cities. Furthermore, within each of the selected schools, we implemented a stratified sampling approach, selecting potential student participants from multiple classes spanning different grades. The collective goal of these strategies was to minimize the biases inherent in convenience sampling. Within each educational institution, class teachers were tasked with distributing the research participation invitation to the students, ensuring their voluntary engagement in either the online or offline surveys. The survey was conducted in a classroom setting to ensure a familiar and structured environment. To standardize the procedure, a trained teacher (serving as the experimenter) provided clear verbal instructions and addressed participants’ questions prior to the survey, ensuring that all students fully understood the response requirements. Subsequently, participants completed the questionnaire independently under the teacher’s supervision, rating each item based on their personal experiences.

For young and middle-aged adults, we employed a snowball sampling technique, leveraging the reach of social media platforms to disseminate recruitment information and invite a wide range of participants to take part in the online survey. Specifically, we targeted the adult population using snowball sampling and utilized WeChat Moments for information dissemination. According to the China Internet Network Information Center (2025), the number of social media users in China has reached 1.101 billion, accounting for 99.3% of internet users and 78.05% of the total population. Social media usage is particularly prevalent among both rural and urban residents. WeChat, as the most popular social media platform in China, boasts an active user base of 1.37 billion, with 75% of its users engaging on WeChat, translating to over 1 billion active users. The extensive reach helps mitigate the risk of excluding rural or less connected populations from our sample. An independent-samples *t*-test in the second split-half sample (*N*_2_ = 1226) revealed no statistically significant difference in AI literacy scores between urban and rural participants at the conventional α = 0.05 level (*t* = 1.90, *p* = 0.06; urban: *n* = 747, *M* = 3.80, *SD* = 0.49; rural: *n* = 479, *M* = 3.72, *SD* = 0.85; *Cohen’s d* = 0.12), suggesting a negligible difference. However, the *p*-value approaches the conventional threshold, indicating that the difference should not be completely dismissed. In addition, to mitigate potential risks of duplicate participation, multiple verification measures were implemented throughout the data collection process. At the recruitment stage, all participant materials clearly indicated that individuals who had previously completed the survey were ineligible for repeated participation. During data processing, additional validation procedures were instituted for respondents who provided identical answers across all questionnaire items. This validation process involved cross-checking demographic information against the survey platform’s automatically recorded geographical data, including the province, city, and full address derived from IP information. For participants exhibiting complete consistency across all identifying information, only the first complete dataset was retained, while subsequent duplicate entries were systematically removed from the final analysis.

We ultimately collected data from 2443 participants (see Table 2), comprising 1144 males and 1299 females. To streamline the analysis process, we employed a random split-half method to divide the total sample into two distinct subsets. The random split was conducted to enable a two-stage validation strategy: item reduction on one half; and reliability and validity testing on the other, following established scale development practices. Sample 1 (*N* = 1217) was designated for item reduction purposes (item discrimination and exploratory factor analysis), whereas Sample 2 (*N* = 1226) was utilized for confirmatory factor analysis, along with assessments of reliability and validity. The strategy of randomly dividing a large sample into two subsets has been previously adopted in scale development research (e.g., Grilo et al., 2010; Ma et al., 2024; Sagherian et al., 2018; Yu & Richardson, 2015), as it simplifies the research workflow. Given the anonymous nature of our survey, particularly when the snowball sampling method was used for the adult population, there was a concern that some individuals might have participated more than once if the data were collected in separate phases. However, it is crucial to maintain separate participant groups for exploratory and confirmatory factor analyses. By conducting a single round of data collection, we mitigated the need for participants to fill out the survey twice. Additionally, conducting multiple rounds of data collection would have increased the workload for schoolteachers, potentially hindering our ability to secure their cooperation. The participant characteristics for the two samples are summarized in Table 2.

### 2.4. Item Reduction

We performed item reduction through item discrimination analysis and exploratory factor analysis. Since the item–total correlation analysis, as one of the prominently used methods in the item reduction process, is more suitable for unidimensional scales, we utilized the critical ratio for high-score versus low-score group differences (greater than 3) to meticulously identify which items would be included in the subsequent exploratory factor analysis (Hair et al., 1998). Prior to conducting the exploratory factor analysis, we first conducted the Kaiser–Meyer–Olkin (KMO) test to determine whether the data were suitable for exploratory factor analysis.

### 2.5. Confirmatory Factor Analysis Strategy

We conducted confirmatory factor analysis to determine whether the scale structure established through exploratory factor analysis was valid. 

### 2.6. Reliability and Validity Testing

The reliability of the Six-Facet AI Literacy Questionnaire was evaluated using Cronbach’s alpha coefficients. To assess the construct validity of the scale, confirmatory factor analysis was conducted. Additionally, we established convergent validity by examining the composite reliability (CR) values, average variance extracted (AVE) values, and correlation coefficients between the scores from the newly developed AI literacy scale and those from an established technology literacy scale, specifically the digital literacy scale (Kang et al., 2023). Furthermore, we examined the heterotrait–monotrait (HTMT) ratio of correlations to assess the discriminant ability among the six dimensions of the scale, thereby confirming the scale’s discriminant validity. Finally, we used academic self-efficacy and academic engagement as criterion variables to examine the criterion-related validity of the Six-Facet Artificial Intelligence Literacy Questionnaire (SFAILQ) in adolescents and university students. Academic engagement was measured using the Utrecht Work Engagement Scale–Student–9 (Schaufeli et al., 2002; Wei et al., 2025), which comprises nine items rated on a 7-point scale (e.g., “I feel strong and vigorous when I’m studying or going to class”). Academic self-efficacy was assessed using the Chinese version of the Academic Self-Efficacy Scale (ASES-C; Zhao et al., 2024), consisting of eight items rated on a 7-point scale (e.g., “I am confident that I can achieve good exam results if I really put my mind to it”).

## 3. Results

The results of item reduction comprise two primary analyses: item discrimination and exploratory factor analysis. Since item-total correlation is more appropriate for unidimensional scales, we exclusively utilize the critical ratio to assess the item discrimination of each individual item. Model fit was evaluated using *χ*^2^, CFI, RMSEA (with 90% CI), and SRMR. Standardized factor loadings and detailed fit statistics are reported in the CFA results. Additionally, Cronbach’s alpha coefficients, composite reliability (CR) values, and the heterotrait–monotrait (HTMT) ratio are presented in the reliability and validity analysis section.

### 3.1. Item Discrimination

Table 3 presents the critical ratio for each item. Participants were ranked on the basis of their total scores, with the top 27% classified as the high-score group and the bottom 27% labelled the low-score group. The scores for each item were subsequently compared between the two groups. Remarkably, eight items either demonstrated nonsignificant differences in scores between the groups or, although significant, had a critical ratio of less than 3. Prior to analysis, all negatively keyed items were reverse-coded so that higher scores consistently reflected higher levels of the measured construct. For reverse-coded items, a negative critical ratio (CR) is expected and indicates good discrimination, because higher-scoring participants tend to endorse the item less strongly before recoding. Items were retained if their CR exceeded an absolute value of 3.00 (or, for reverse-coded items, if the absolute CR was ≥ 3.00). Item 9 had an absolute CR of 9.23, indicating strong discriminatory power, and was therefore retained at the discrimination stage. As a result, we removed a total of eight items (items 6, 7, 8, 16, 29, 30, 34, and 56), leaving a final count of 56 items.

### 3.2. Exploratory Factor Analysis

Before conducting the exploratory factor analysis, we conducted a parallel analysis (Horn, 1965) to determine the optimal number of factors. Parallel analysis involves comparing the eigenvalues derived from the actual dataset with those obtained from randomly generated datasets, thereby providing a more reliable criterion for factor extraction. The results suggested a solution comprising seven factors. Consequently, we set the number of factors at seven for the subsequent factor analysis. Exploratory factor analysis was performed using principal axis factoring (PAF) with a Promax rotation. The KMO value was 0.98 for the 56-item scale. The indices of Bartlett’s test of sphericity were as follows: *χ*^2^ = 61,255.87, *df* = 1540, *p* < 0.001. These results showed that the correlation matrix was suitable for exploratory factor analysis. The initial results (see Table 4) revealed that seven factors with eigenvalues exceeding 1 were extracted, accounting for 69.11% of the total variance. However, numerous items exhibited substantial cross-loadings. Specifically, the initial seven-factor solution extracted five items (Items 27, 28, 37, 44, and 54) that loaded primarily onto the seventh factor. These five items did not form a conceptually interpretable independent dimension; rather, they primarily reflected contextual AI usage scenarios that substantially overlapped with three other theoretical dimensions. Moreover, all five items showed prominent cross-loadings, with primary-secondary loading differences below 0.30, indicating poor discriminant validity and a lack of structural robustness for that factor. Consequently, we removed these items based on the established criteria, in accordance with standard factor analysis practices.

While the parallel analysis suggested seven factors, close inspection revealed that the seventh factor primarily captured item-specific variance and content redundancy rather than a substantively meaningful construct. We therefore proceeded with iterative item deletions based on established criteria (commonality < 0.40, primary loadings < 0.40, and cross-loading differences < 0.30). On the basis of these criteria, we systematically excluded items 9, 42, 41, 31, 32, 10, 11, 19, 20, 22, 21, 24, 23, 44, 37, 40, 54, 51, 52, 53, 17, 55, 57, and 61. The four items designed to assess negative affective experiences were excluded during the item reduction phase, as they failed to meet pre-established psychometric criteria in both item discrimination analysis and exploratory factor analysis (EFA). Descriptive statistics for these items (see Table 5) revealed low means (*M* = 1.94–2.22 on a 5-point scale), relatively small standard deviations (*SD* = 0.94–1.15), and positive skewness (0.46–0.77), indicating that participants rarely endorsed negative affective states. The response distributions exhibited a pronounced floor effect: across all four items, the proportion of participants choosing the lowest two response options (“Strongly disagree” or “Disagree”) ranged from 65.0% to 72.3%, while the highest two options (“Agree” or “Strongly agree”) were selected by fewer than 15% of respondents. Furthermore, these items showed poor discriminative power, with corrected item-total correlations below 0.30 and EFA factor loadings lower than 0.40. Taken together, these distributional and psychometric limitations warranted the removal of these items from subsequent analyses.

The exploratory factor analysis produced a six-facet scale comprising 32 items, which explained 73.76% of the total variance (see Table 6). Factor 1, consisting of six items, was designated “ethical norms.” Factor 2, also comprising six items, was labelled “self-development.” Factor 3, with five items, was identified as “affective experiences.” Factor 4, containing six items, was classified as “cognitive evaluation.” Factor 5, which included five items, was named “usage skills,” whereas Factor 6, comprising four items, was termed “responsible use.” The scientifically grounded item reduction process naturally led to the emergence of a more robust six-facet solution. Our approach therefore was not a post hoc “fit to the desired model,” but rather an iterative process guided by both theoretical alignment and standard psychometric criteria. No theoretically unique content was lost during the process, and that the final six-factor model was ultimately adopted because it provided the most parsimonious and theoretically coherent interpretation of the data. The complete set of measurement items for all factors is available in the Appendix B.

### 3.3. Confirmatory Factor Analysis

**Figure 1:**
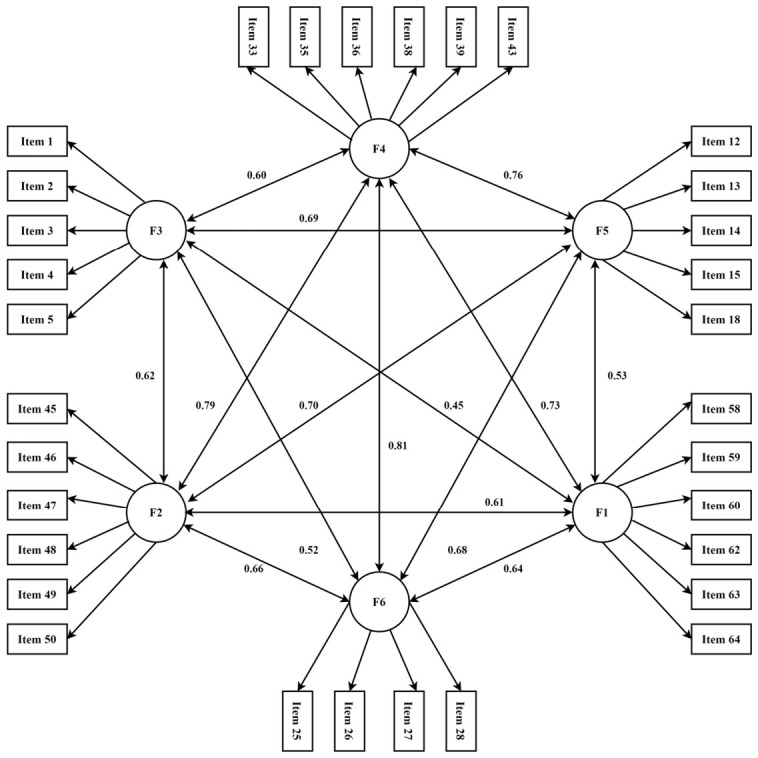
Confirmatory factor analysis of the Six-Facet AI Literacy Questionnaire (SFAILQ). Note. *N* = 1226. F1 = Ethical norms. F2 = Self-development. F3 = Affective experiences. F4 = Cognitive evaluation. F5 = Usage skills. F6 = Responsible use.

Confirmatory factor analysis (see Figure 1) was conducted to evaluate the construct validity of the Six-Facet AI Literacy Questionnaire (SFAILQ). The standardized parameter estimates for all the items were statistically significant (*p* < 0.01), with factor loadings exceeding 0.60 for each item (see Table 7). The six-facet model demonstrated a marginal but acceptable fit to the data: *χ*^2^/*df* = 6.06, CFI = 0.93, RMSEA = 0.06 (90% CI [0.056, 0.064]), and SRMR = 0.05. While the *χ*^2^/*df* value exceeded the conventional threshold of 5.0, the deviation is likely attributable to the large sample size (*N* = 1226), which is known to inflate the chi-square statistic (Bentler & Bonett, 1980). The remaining fit indices all met or exceeded recommended thresholds for acceptable model fit (CFI ≥ 0.90, RMSEA ≤ 0.08, SRMR ≤ 0.08; Hu & Bentler, 1999), supporting the adequacy of the six-facet structure. Collectively, these results indicate that the Six-Facet AI Literacy Questionnaire has good construct validity.

### 3.4. Measurement Invariance Across Age Groups

To examine whether the SFAILQ measures the same construct equivalently across the three age groups (adolescents: 12–17 years; young adults: 18–40 years; middle-aged adults: 41–60 years), we conducted a series of multi-group confirmatory factor analyses (MG-CFA). Following established procedures (Cheung & Rensvold, 2002; Chen, 2007), we tested three progressively constrained models: configural invariance, metric invariance, and scalar invariance. Model fit was evaluated using the comparative fit index (CFI) and the root mean square error of approximation (RMSEA). Invariance was supported if the change in CFI (ΔCFI) was ≤0.01 and the change in RMSEA (ΔRMSEA) was ≤0.015 between adjacent nested models (Cheung & Rensvold, 2002; Chen, 2007).

As shown in Table 8, the configural model demonstrated acceptable fit, indicating that the same six-factor structure holds across the three age groups. The metric model also showed good fit, with ΔCFI = 0.005 and ΔRMSEA = 0.005, both within the recommended thresholds, supporting equal factor loadings across groups. The scalar model yielded ΔCFI = 0.008 and ΔRMSEA = 0.007, also within the acceptable range. While the scalar invariance test was successful, the ΔCFI value approached the conventional threshold of 0.01, suggesting that scalar invariance may be somewhat less robust than metric invariance. This warrants cautious interpretation of mean comparisons across age groups, although the overall pattern of results supports the psychometric justification for such comparisons. Taken together, these results support scalar invariance across the three age groups, indicating that the SFAILQ measures the same underlying construct equivalently across adolescents, young adults, and middle-aged adults.

### 3.5. Reliability and Validity Analysis

Table 9 and Table 10 display the results of the reliability and validity analysis of the SFAILQ. The internal consistency coefficients for the six dimensions of the AI literacy scale, as well as for the overall scale, all exceed 0.85, significantly surpassing the acceptable threshold of 0.70. Thus, the scale we developed demonstrates good reliability.

The composite reliability (CR) values for the six dimensions and the overall scale exceed the acceptable threshold of 0.70, whereas the average variance extracted (AVE) values for these dimensions and the overall scale surpass the acceptable threshold of 0.50. The Fornell-Larcker comparison (Fornell & Larcker, 1981) is presented in Table 6. As shown, the square root of the AVE for each dimension (√AVE) ranges from 0.74 to 0.84, and all √AVE values exceed the corresponding inter-factor correlations, satisfying the criterion for discriminant validity. Additionally, the scores for both the individual dimensions and the overall AI literacy scale are significantly correlated with the scores of the digital literacy scale, with correlation coefficients ranging from 0.49 to 0.68. Collectively, these findings suggest that our scale has strong convergent validity.

The correlation coefficients among the six dimensions of the SFAILQ ranged from 0.43 to 0.72. Although some dimensions displayed correlations exceeding 0.70, none reached or surpassed 0.80, indicating that there are no excessively high correlations present. Furthermore, the heterotrait–monotrait (HTMT) ratio of the correlations among the six factors did not exceed 0.80, aligning with the recommended threshold of not exceeding 0.85. These values are relatively high but still below the conventional threshold of 0.85 (Kline, 2015), supporting discriminant validity. The relatively high correlations are theoretically plausible, as all dimensions collectively measure the broader construct of AI literacy, and some conceptual overlap is inherent in multidimensional constructs. Overall, these findings suggest that the scale has good discriminant validity.

The criterion-related validity analysis revealed several meaningful patterns. All six dimensions and the total score of the SFAILQ were significantly positively correlated with students’ academic self-efficacy, with the usage skills dimension showing the strongest correlation. Similarly, all six dimensions and the total score were significantly positively correlated with students’ academic engagement, where the responsible use dimension demonstrated the strongest association. Extending these findings to the working population, all six dimensions and the total score also correlated significantly positively with employees’ work self-efficacy, with the self-development dimension showing the strongest correlation. 

## 4. Discussion

The rapid advancement of AI has made AI literacy a prominent area of interest across various disciplines. However, the limited availability of measurement tools poses a significant challenge that may hinder researchers’ ability to conduct studies effectively. Currently, over thirty distinct tools exist for assessing digital literacy (Nguyen & Habók, 2024; Oh et al., 2021), with new scales continuing to emerge in recent years (e.g., Avinç & Doğan, 2024; Hwang et al., 2023; Reddy et al., 2023). The availability of a diverse range of measurement tools has greatly advanced theoretical research and educational practices in digital literacy. Consequently, in today’s AI era, expanding the concept of AI literacy and developing AI literacy scales are of substantial theoretical and practical importance.

Building on previous research, we defined AI literacy from an integrative perspective. Guided by the six-facet model, we developed an AI literacy questionnaire structured around its six core factors. The dimensions of the scale are as follows: the affective experiences dimension consists of 5 items, the usage skills dimension contains 5 items, the cognitive evaluation dimension includes 6 items, the ethical norms dimension consists of 6 items, the responsible use dimension contains 4 items, and the self-development dimension comprises 6 items. Overall, the Six-Facet AI Literacy Questionnaire comprises 32 items, demonstrating strong reliability and validity, thus rendering it highly effective for measuring AI literacy in adolescents, young adults and midlife adults.

Existing measurement tools (Ng et al., 2024; B. Wang et al., 2023; Zhou et al., 2024) provide a solid foundation for our Six-Facet AI Literacy Questionnaire. In the six-facet model, the four dimensions (i.e., affective experiences, usage skills, cognitive evaluation, and ethical norms) correspond with the dimensions of established measurement tools. Thus, our instrument shares commonalities with previous scales, effectively capturing the core factors of AI literacy emphasized in prior research. The similarities are particularly evident in the dimensions of affective experiences, cognitive evaluation, and ethical norms. Importantly, during the item reduction procedure, we removed four items assessing negative affective experiences, despite our initial intention to categorize affective experiences into positive and negative categories. At the initial stage of scale development, we were eager to incorporate aspects of negative affective experiences. Furthermore, given that existing AI literacy scales overlook negative affective experiences, we aimed to make the inclusion of such experiences a distinctive feature of our scale. Unfortunately, during the analysis of item discrimination for the project, the three items associated with negative affective experiences failed to demonstrate satisfactory discrimination. Additionally, an extra item was removed from the exploratory factor analysis because of poor factor loading. Consequently, during the item reduction phase, we were compelled to eliminate all four items pertaining to negative affective experiences. One plausible explanation for the inadequate item discrimination and factor structure concerning negative affective experiences could be that individuals with high AI literacy tend to experience negative emotions less frequently in AI interactions, whereas those with low AI literacy, owing to their limited engagement, similarly encounter fewer negative emotions. In other words, regardless of whether individuals possess high or low AI literacy, their negative affective experiences in the context of AI are not prevalent. Thus, negative emotional experiences cannot effectively distinguish between various levels of AI literacy. The absence of negative affective experiences in existing AI literacy scales (e.g., B. Wang et al., 2023; Laupichler et al., 2023a; Laupichler et al., 2023b; Zhou et al., 2024) is likely attributed to the fact that these experiences do not accurately reflect differences in AI literacy levels. Additionally, a notable distinction of our scale lies in the fact that AI usage skills encompass both objective skill levels (actual behaviour) and subjective skill assessments (self-efficacy). As previously highlighted by researchers (Chu et al., 2018; Rath et al., 2004), the assessment of problem-solving abilities similarly emphasizes both objective capabilities and subjective efficacy. At its core, AI literacy denotes the capacity to use AI tools effectively to address practical challenges. Thus, measuring both objective skills and subjective efficacy is imperative to characterize an individual’s proficiency in AI usage more comprehensively.

One innovation of the SFAILQ is the incorporation of responsible use as a core factor. There are a total of four questions specifically designed to measure responsible use. AI is becoming increasingly integrated into various aspects of daily life. However, the excessive use of any technology can lead to a variety of negative consequences. Specifically, an overreliance on AI may diminish an individual’s cognitive ability, thereby undermining their capacity to think independently, deeply, and solve complex problems (Naseer et al., 2025; Parsakia, 2023; Zhai et al., 2024). On the other hand, resisting the use of AI undoubtedly alienates one from technological advancement and fails to demonstrate AI literacy (Faruqe et al., 2022). The responsible use of AI technology enables individuals to enhance their skills and address challenges while minimizing excessive dependence and mitigating potential negative consequences. Among the four items of the responsible use dimension, two measure the ability to control the usage intensity, whereas the other two assess whether a person is overly reliant on AI to the extent of even completely delegating their tasks to AI, thereby demonstrating a lack of responsibility towards oneself. The first two items measure usage intensity to indirectly reflect the “responsible use” principle in DigCompEdu (Redecker, 2017) that safeguards against negative impacts on physical, psychological and social wellbeing—since clearly higher usage intensity tends to produce more adverse effects. The last two items, which emphasize avoiding irresponsible AI use and not delegating tasks requiring human accountability entirely to AI, directly embody DigCompEdu’s (Redecker, 2017) core notion of using digital technologies safely and responsibly. Thus, these four items collectively reflect the concept of responsible use.

Another significant innovation of our Six-Facet AI Literacy Questionnaire is the inclusion of self-development as a core dimension. Six items were specifically designed to measure self-development. On the one hand, the concept of self-development encompasses individuals continually enhancing their theoretical knowledge and professional skills in AI; on the other hand, it involves utilizing these increasingly advanced AI skills to foster personal growth, such as improving quality of life. In the realm of digital literacy research, the importance of self-development has already been highlighted by several scholars (W. J. Wang et al., 2020). There is a growing consensus that leveraging the internet to support self-development is a vital aspect of internet literacy (Berezhna et al., 2021; Ma et al., 2024; W. J. Wang et al., 2020). Focusing solely on internet usage skills while overlooking the developmental outcomes that arise from skill application risks transforming the discussion into an overly theoretical analysis, severing the connection between capabilities and practical activities. By emphasizing self-development within the context of AI literacy, we contribute to a dynamic understanding of AI literacy as a continuously evolving factor, measuring both current competencies and future potential.

Furthermore, the SFAILQ demonstrated satisfactory reliability and validity among adolescents, young adults, and midlife adults in the present study. Existing tools for measuring AI literacy have focused predominantly on either adolescents (Ng et al., 2024) or adults (e.g., Laupichler et al., 2023a; Laupichler et al., 2023b; Zhou et al., 2024), leading to a significant gap in the comparative assessment of AI literacy between these two groups. As a result, no scale exists that can concurrently evaluate AI literacy across adolescents, young adults, and midlife adults. The use of various tools to compare AI literacy between adolescents and adults may lead to inaccurate conclusions when direct comparisons are drawn, owing to the variability inherent in the measurement instruments. However, contrasting the results between adolescents and adults using the same scale has the potential to deepen our understanding of how individuals at different developmental stages theoretically comprehend and practically apply AI technology. The SFAILQ is specifically designed for diverse demographic groups, particularly adolescents, young adults, and midlife adults, to yield rigorous and accurate results for future analyses of AI literacy. The establishment of scalar invariance across adolescents, young adults, and middle-aged adults is a key psychometric strength of the SFAILQ. It provides empirical support for the claim that scores obtained from different age groups can be meaningfully compared, thereby reinforcing the utility of the instrument as a developmentally versatile tool for assessing AI literacy across the lifespan. In future research, our scale has the potential to enable meaningful comparisons of overall AI literacy levels among adolescents, young adults, and middle-aged adults, as well as to clarify the differences in their proficiency across various intrinsic components of AI literacy.

Notably, all six dimensions and the total score of the SFAILQ were significantly and positively correlated with academic self-efficacy and academic engagement among students, as well as with work self-efficacy among employees. Moreover, distinct patterns emerged across the three criterion variables: usage skills showed the strongest correlation with academic self-efficacy; responsible use demonstrated the highest correlation with academic engagement; and self-development showed the strongest correlation with work self-efficacy. The fact that all six dimensions simultaneously exhibited positive correlations with both academic self-efficacy and academic engagement indicates that AI literacy is a comprehensive, cross-task, and cross-situational competence. It not only supports basic operational skills in learning but also profoundly influences learners’ intrinsic motivation and long-term willingness to learn. The distinct pattern of the strongest correlations, namely between usage skills and self-efficacy and between responsible use and engagement, further demonstrates that the different dimensions of the SFAILQ capture distinct facets of psychological functioning. According to Bandura’s (1997) social cognitive theory, mastery experiences are the most powerful source of self-efficacy. Hands-on, direct, and successful operational experiences most effectively strengthen the belief that “I can do it” (Bandura, 1997). The usage skills dimension of the SFAILQ directly measures students’ concrete operational competencies in AI-based learning scenarios. When learners achieve a learning goal or complete a task through practical application, their success experience is directly and robustly internalised as part of their self-efficacy. Individuals who can independently use AI to accomplish tasks are more likely than those who merely understand AI concepts to believe in their ability to meet future learning challenges. On the other hand, academic engagement differs from self-efficacy. Engagement emphasizes the emotional, cognitive, and behavioural commitment to investing effort in learning (Quin et al., 2017). Responsible use repositions AI from an external tool to an integral part of learners’ self-regulation or self-control. Responsible use is not a checklist but a mindset. Once internalised, it can provide a stable guide for sustained behaviour. Furthermore, self-development captures individuals’ motivation and capacity to proactively learn and adapt to new technological demands, which is particularly relevant to the workplace, where continuous upskilling and adaptation to AI tools are increasingly critical for maintaining competence and confidence (Rukadikar & Khandelwal, 2024; Zahrani, 2025). Taken together, these findings support the criterion-related validity of the SFAILQ across age groups and criterion domains, and underscore the differentiated contributions of its specific facets to varied functional outcomes.

From a practical perspective, the considerable potential of our SFAILQ in educational practice warrants particular emphasis. First, for specific age groups, our SFAILQ may not only describe the overall development status of individuals’ AI literacy, but also compare whether there are significant differences among the six specific dimensional factors. The results from our scale may thereby offer targeted developmental recommendations for particular demographics. For example, for upper-secondary or vocational students, their limited age and AI experience may lead to lower scores in practical skills; while their underdeveloped self-control ability may result in excessive reliance on AI, leading to lower scores in responsible use. Our scale enables the assessment of both overall AI literacy and dimensional-specific performance. It can reveal basic AI literacy levels, identify developmental gaps, and support the development of practical AI skills while preventing over-reliance on AI among students. Similarly, for educators’ professional development, our SFAILQ can to some extent perform functions similar to SelfieforTeachers (developed based on DigCompEdu)—supporting teachers to create personalized development plans. We can first use the six-dimensional assessment of SFAILQ for initial self-evaluation among educators, generating personalized AI literacy profiles to identify strengths and weaknesses for targeted training. Moreover, SFAILQ can also serve as an evaluation tool after training to compare whether significant changes occur in the AI literacy profiles before and after the training. Second, for different age groups, our SFAILQ can not only compare their overall AI literacy levels but also examine differences across the six specific dimensions of AI literacy. The results can then guide educational institutions in developing corresponding training programs. For example, in institutional needs assessments to inform training and curriculum design, educational institutions can design age-specific courses based on the detailed findings of age-related differences in AI literacy, identifying distinct focal dimensions as core training components for different age groups. In summary, our scale functions as both an initial assessment tool and a guiding framework for instructional design and professional development programs, as well as an evaluation instrument to measure effectiveness following courses or intervention training. Despite demonstrating substantial applicability in educational contexts, the SFAILQ nevertheless requires comprehensive validation in larger, more diverse cohorts before any real-world deployment can be justified. In our future research, we will undertake empirical studies to investigate the practical application of SFAILQ at different stages of educational practice among diverse age groups.

## 5. Limitations

The present study has several limitations. First, the removal of all four negative affective items narrowed the affective dimension to positive experiences only, which deviates from our original theoretical intent to capture both positive and negative emotional responses to AI. The removal of these items was empirically justified, as they showed highly skewed distributions, low means, and limited variability, indicating that negative affective experiences were rarely endorsed by our participants. Nevertheless, the conceptual limitation remains: the SFAILQ’s affective dimension does not currently cover negative affective experiences, such as anxiety, technostress, or frustration. Future research should consider developing complementary measures specifically targeting negative affective responses to AI to capture the full spectrum of emotional engagement with AI technologies. Second, while the sample covered a broad age spectrum from 12 to 60 years, the vast majority of participants were adults between 18 and 40, which warrants special caution in the interpretation and generalization of the findings. The applicability of the SFAILQ for children and elderly populations still needs to be validated. For children raised in the era of AI, the influence of AI technology may be particularly profound. Furthermore, research reveals that the frequency of AI utilization among older adults is steadily increasing, and this increased engagement with AI has considerable effects on cognitive functioning and mental health within this demographic (Lee et al., 2022; Park & Kim, 2022). While conducting surveys with young children and individuals over 60 years of age is methodologically feasible through approaches such as one-on-one interviews, our preliminary research encountered substantial practical limitations. The initial version of the scale, containing an excessive number of items, proved to be impractical because of the considerable time and resources required for individual administration, ultimately prolonging the research timeline. These challenges proved insurmountable in our initial study. In future studies, we plan to utilize the validated version of the SFAILQ with fewer items to conduct surveys among children and elderly individuals, aiming to establish the reliability and validity of our scale across a broader age range. Third, several limitations concerning sampling and generalizability should be noted. The use of convenience and snowball sampling, combined with mixed online and offline administration for adolescents, may limit the representativeness of the sample. The difference in recruitment frames may have introduced sampling-related biases that could affect the comparability of the three age groups. Although we included age as a grouping variable in the measurement invariance analysis and controlled for demographic variables where applicable, the potential influence of recruitment-related heterogeneity should be considered when interpreting the cross-age comparisons. Future studies may benefit from employing more consistent recruitment strategies across age groups to strengthen the comparability of findings. Additionally, the sample was entirely Chinese, meaning that the cross-cultural generalizability of the SFAILQ remains to be established. Future research should therefore test the factorial structure and measurement properties of the SFAILQ in diverse cultural and national contexts. Furthermore, although we included a comparison between urban and rural participants, the group sizes and effect sizes should be interpreted with caution given the marginal significance and the sampling constraints. Together, these considerations suggest that the present findings provide an important initial validation of the SFAILQ, but further replication in more representative and diverse samples is warranted. Fourth, although splitting a single dataset into random halves is a commonly accepted practice in scale development, it does not provide the same level of cross-validation as replication in an entirely independent sample. Future research should therefore replicate the SFAILQ’s factor structure in independent samples to further confirm its stability. Finally, one limitation concerns the high internal consistency observed for the total AI literacy score (α = 0.97, CR = 0.99). While these values attest to the strong reliability of the scale, they may also suggest some degree of item redundancy, a possibility partly attributable to the relatively large number of items (32 items across six dimensions), as Cronbach’s α is known to increase with scale length. Importantly, the six dimensions demonstrated good discriminant validity (as indicated by the Fornell-Larcker comparison), suggesting that the scale captures distinct facets of AI literacy despite the high overall reliability. Future research may consider developing a shortened version of the SFAILQ to reduce respondent burden and further examine whether item reduction compromises construct coverage.

## 6. Conclusions

Building on the six-facet model of AI literacy, we developed the Six-Facet Artificial Intelligence Literacy Questionnaire (SFAILQ), a 32-item instrument comprising six dimensions: affective experiences (5 items), usage skills (5 items), cognitive evaluation (6 items), ethical norms (6 items), responsible use (4 items), and self-development (6 items). All six dimensions and the total score of the SFAILQ were significantly positively correlated with academic self-efficacy, with the usage skills dimension demonstrating the strongest correlation. Similarly, both the six dimensions and the total score were significantly positively correlated with academic engagement, with the responsible use dimension showing the highest correlation. The SFAILQ demonstrated satisfactory reliability and validity across adolescent, young adult, and middle-aged populations. Theoretically, the present study advances our understanding of AI literacy and encourages future research to explore the construct from a broader and more integrative perspective. Practically, the SFAILQ enriches the existing toolkit of AI literacy measures, offering a convenient and effective instrument for subsequent research. In conclusion, the SFAILQ is well-positioned to make a meaningful contribution to both theoretical research and educational practice concerning AI literacy across diverse age groups, including adolescents, young adults, and midlife adults.

## Figures and Tables

**Table 1:** Comparison of theoretical frameworks for AI literacy.

Key Points	ABCE Model	KSAVE Model		Six-Facet Model
Core factors of the target model	Affective learning Behavioural learning Cognitive learning Ethical learning	Knowledge Skills Attitudes Values Ethics		Affective experiences Usage skills Cognitive evaluation Ethical norms Responsible use Self-development
Measurements based on the target model	AL Literacy Questionnaire (Ng et al., 2024)	Artificial Intelligence Literacy Scale (B. Wang et al., 2023)	AI Literacy Competency Measurement Scale (Zhou et al., 2024)	Six-Facet AI Literacy Questionnaire
	A four-factor scale provides support for the proposed model.	A four-factor scale lacks support for the proposed model.	A four-factor scale lacks support for the proposed model.	
Age range of the scale based on the target model	12 to 17 years old	17 to 65 years old	University Students (Mage = 21)	12 to 60 years old (adolescents, young adults, and midlife adults)

**Table 2:** Characteristics of the participants.

Characteristics		Number (Percentage)	
		Sample 1	Sample 2
Sex	Male	566 (46.5%)	578 (47.1%)
	Female	651 (53.5%)	648 (52.9%)
Age	Adolescents (aged 12 to 17 years)	385 (31.6%)	391 (31.9%)
	Young adults (aged 18 to 40 years)	551 (45.3%)	570 (46.5%)
	Midlife adults (aged 41 to 60 years)	281 (23.1%)	265 (21.6%)
Developmental stage	Middle school students	251 (20.6%)	263 (21.5%)
	High school students	169 (13.9%)	191 (15.6%)
	University students	433 (35.6%)	445 (36.3%)
	Working population	364 (29.9%)	327 (26.7%)

Note. *N_sample_*_1_ = 1217, *N_sample_*_2_ = 1226.

**Table 3:** Results of the item discrimination test.

Items	*t* (Critical Ratio)	*p*	Items	*t* (Critical Ratio)	*p*
Item 1	21.17	<0.001	Item 33	24.46	<0.001
Item 2	19.66	<0.001	Item 34	1.22	0.22
Item 3	21.06	<0.001	Item 35	22.93	<0.001
Item 4	20.84	<0.001	Item 36	28.74	<0.001
Item 5	22.88	<0.001	Item 37	28.63	<0.001
Item 6	−0.82	0.41	Item 38	24.74	<0.001
Item 7	−2.31	0.02	Item 39	20.41	<0.001
Item 8	−1.65	0.10	Item 40	16.94	<0.001
Item 9	−9.23	<0.001	Item 41	30.50	<0.001
Item 10	17.87	<0.001	Item 42	30.09	<0.001
Item 11	25.07	<0.001	Item 43	19.99	<0.001
Item 12	27.02	<0.001	Item 44	22.54	<0.001
Item 13	28.50	<0.001	Item 45	27.96	<0.001
Item 14	27.95	<0.001	Item 46	27.51	<0.001
Item 15	24.58	<0.001	Item 47	31.99	<0.001
Item 16	1.74	0.08	Item 48	29.62	<0.001
Item 17	26.63	<0.001	Item 49	30.31	<0.001
Item 18	23.34	<0.001	Item 50	31.20	<0.001
Item 19	29.23	<0.001	Item 51	23.74	<0.001
Item 20	28.38	<0.001	Item 52	26.81	<0.001
Item 21	27.70	<0.001	Item 53	29.04	<0.001
Item 22	25.77	<0.001	Item 54	32.65	<0.001
Item 23	25.15	<0.001	Item 55	28.74	<0.001
Item 24	27.40	<0.001	Item 56	−0.29	0.78
Item 25	25.92	<0.001	Item57	20.18	<0.001
Item 26	26.96	<0.001	Item 58	26.82	<0.001
Item 27	26.31	<0.001	Item 59	28.46	<0.001
Item 28	25.57	<0.001	Item 60	28.00	<0.001
Item 29	2.11	0.04	Item 61	26.41	<0.001
Item 30	0.64	0.52	Item 62	22.54	<0.001
Item 31	15.10	<0.001	Item 63	24.29	<0.001
Item 32	15.90	<0.001	Item 64	24.63	<0.001

**Table 4:** Initial results of the exploratory factor analysis.

Items (Commonality)	Factor 6	Factor 5	Factor 3	Factor 7	Factor 4	Factor 1	Factor 2
Item 1 (0.73)	0.77						
Item 2 (0.74)	0.77						
Item 3 (0.79)	0.78						
Item 4 (0.77)	0.75						
Item 5 (0.76)	0.72						
Item 9 (0.42)				−0.54			
Item 10 (0.65)		0.69					
Item 11 (0.76)		0.72					
Item 12 (0.75)		0.70					
Item 13 (0.77)		0.68					
Item 14 (0.76)		0.67					
Item 15 (0.65)		0.54					
Item 17 (0.63)			0.44				
Item 18 (0.64)		0.42	0.51				
Item 19 (0.74)			0.61				
Item 20 (0.71)			0.61				
Item 21 (0.73)			0.63				
Item 22 (0.70)			0.61				
Item 23 (0.72)			0.65				
Item 24 (0.74)			0.66				
Item 25 (0.72)			0.67				
Item 26 (0.71)			0.63				
Item 27 (0.67)			0.57				0.45
Item 28 (0.62)			0.52				0.45
Item 31 (0.64)				0.66			
Item 32 (0.67)				0.67			
Item 33 (0.57)					0.41		
Item 35 (0.59)					0.43		
Item 36 (0.64)					0.50		
Item 37 (0.67)					0.53		0.40
Item 38 (0.65)					0.60		
Item 39 (0.74)					0.75		
Item 40 (0.67)					0.74		
Item 41 (0.67)					0.49	0.46	
Item 42 (0.72)					0.48	0.50	
Item 43 (0.53)					0.51		
Item 44 (0.61)					0.57		0.44
Item 45 (0.68)						0.68	
Item 46 (0.72)						0.72	
Item 47 (0.75)						0.74	
Item 48 (0.74)						0.72	
Item 49 (0.78)						0.75	
Item 50 (0.76)						0.74	
Item 51 (0.62)						0.63	
Item 52 (0.77)						0.77	
Item 53 (0.76)						0.77	
Item 54 (0.70)						0.64	0.41
Item 55 (0.68)							0.68
Item 57 (0.51)							0.60
Item 58 (0.66)							0.70
Item 59 (0.83)							0.85
Item 60 (0.75)							0.79
Item 61 (0.63)							0.69
Item 62 (0.68)							0.77
Item 63 (0.75)							0.82
Item 64 (0.71)							0.78
Eigenvalues	4.42	4.84	6.50	1.89	4.84	8.41	7.83
variance explained (%)	7.89	8.64	11.61	3.34	8.64	15.01	13.98

**Table 5:** Descriptive statistics of the four negative affective items.

Items	Mean	SD	Skewness
Item 6	2.09	0.94	0.56
Item 7	2.18	0.96	0.46
Item 8	2.22	1.15	0.50
Item 9	1.94	0.98	0.77

**Table 6:** Final results of the exploratory factor analysis.

Items (Commonality)	Factor 3	Factor 5	Factor 6	Factor 4	Factor 2	Factor 1
Item 1 (0.74)	0.79					
Item 2 (0.74)	0.78					
Item 3 (0.80)	0.81					
Item 4 (0.78)	0.79					
Item 5 (0.77)	0.76					
Item 12 (0.76)		0.71				
Item 13 (0.83)		0.75				
Item 14 (0.82)		0.75				
Item 15 (0.69)		0.67				
Item 18 (0.61)		0.51				
Item 25 (0.77)			0.70			
Item 26 (0.82)			0.73			
Item 27 (0.79)			0.72			
Item 28 (0.73)			0.67			
Item 33 (0.61)				0.56		
Item 35 (0.65)				0.59		
Item 36 (0.66)				0.56		
Item 38 (0.68)				0.64		
Item 39 (0.71)				0.74		
Item 43 (0.55)				0.59		
Item 45 (0.74)					0.73	
Item 46 (0.77)					0.76	
Item 47 (0.81)					0.78	
Item 48 (0.78)					0.76	
Item 49 (0.78)					0.75	
Item 50 (0.75)					0.73	
Item 58 (0.67)						0.69
Item 59 (0.84)						0.85
Item 60 (0.76)						0.79
Item 62 (0.70)						0.77
Item 63 (0.78)						0.84
Item 64 (0.75)						0.80
Eigenvalues	4.15	3.30	2.94	3.38	4.84	5.00
variance explained (%)	12.98	10.31	9.17	10.56	15.11	15.63

**Table 7:** Factor loadings in CFA.

Dimensions	Items	Factor Loadings
Affective experiences	Item 1	0.80
	Item 2	0.84
	Item 3	0.88
	Item 4	0.87
	Item 5	0.82
Usage skills	Item 12	0.84
	Item 13	0.91
	Item 14	0.88
	Item 15	0.76
	Item 18	0.69
Responsible use	Item 25	0.84
	Item 26	0.85
	Item 27	0.82
	Item 28	0.80
Cognitive evaluation	Item 33	0.71
	Item 35	0.72
	Item 36	0.81
	Item 38	0.76
	Item 39	0.75
	Item 43	0.66
Self-development	Item 45	0.83
	Item 46	0.80
	Item 47	0.85
	Item 48	0.83
	Item 49	0.83
	Item 50	0.86
Ethical norms	Item 58	0.80
	Item 59	0.87
	Item 60	0.89
	Item 62	0.81
	Item 63	0.83
	Item 64	0.80

**Table 8:** Measurement invariance across adolescents, young adults, and midlife adults.

Model	*χ*^2^/*df*	CFI	RMSEA	SRMR	ΔCFI	ΔRMSEA
Configural	4.79	0.953	0.049	0.041		
Metric	5.12	0.948	0.054	0.046	0.005	0.005
Scalar	5.69	0.940	0.061	0.052	0.008	0.007

Note. ΔCFI and ΔRMSEA are computed relative to the previous model (Metric vs. Configural; Scalar vs. Metric). Invariance is supported if ΔCFI ≤ 0.01 and ΔRMSEA ≤ 0.015.

**Table 9:** Reliability and validity analysis of the SFAILQ.

Variables	α	CR	AVE	1	2	3	4	5	6	7
1. Affective experiences	0.92	0.92	0.71	√AVE = 0.84	0.70	0.59	0.47	0.54	0.61	—
2. Usage skills	0.91	0.91	0.67	0.64 ***	√AVE = 0.82	0.78	0.55	0.69	0.73	—
3. Cognitive evaluation	0.87	0.88	0.54	0.53 ***	0.70 ***	√AVE = 0.74	0.71	0.79	0.79	—
4. Ethical norms	0.93	0.93	0.70	0.43 ***	0.51 ***	0.65 ***	√AVE = 0.84	0.65	0.61	—
5. Responsible use	0.90	0.90	0.68	0.48 ***	0.63 ***	0.72 ***	0.59 ***	√AVE = 0.83	0.65	—
6. Self-development	0.93	0.93	0.69	0.57 ***	0.67 ***	0.72 ***	0.57 ***	0.60 ***	√AVE = 0.83	—
7. AI literacy	0.97	0.99	0.66	0.74 ***	0.84 ***	0.88 ***	0.78 ***	0.80 ***	0.85 ***	—
8. Digital literacy	0.83	0.83	0.55	0.49 ***	0.58 ***	0.68 ***	0.59 ***	0.60 ***	0.63 ***	0.68 ***

Note. CR = composite reliability; AVE = average variance extracted. The diagonal values represent the square roots of the AVE for each of the six dimensions. Diagonal √AVE values are reported for the six dimensions only. The total AI literacy score (row 7) is a composite score. Digital literacy was included as a criterion variable and is not part of the six-factor structure; thus, its √AVE is not reported on the diagonal. The coefficients below the diagonal are the inter-factor Pearson correlations among the six dimensions. The coefficients above the diagonal are the heterotrait–monotrait (HTMT) ratios. *** *p* < 0.001.

**Table 10:** Criterion-related validity test.

Variables	Affective Experiences	Usage Skills	Cognitive Evaluation	Ethical Norms	Responsible Use	Self-Development	AI Literacy
1. Academic self-efficacy	0.21 ***	0.56 ***	0.40 ***	0.32 ***	0.45 ***	0.41 ***	0.49 ***
2. Academic engagement	0.27 ***	0.47 ***	0.44 ***	0.36 ***	0.59 ***	0.51 ***	0.56 ***
3. Work self-efficacy	0.26 ***	0.50 ***	0.38 ***	0.33 ***	0.41 ***	0.57 ***	0.44 ***

Note. *** *p* < 0.001.

## Data Availability

The data are not publicly available due to privacy and ethical restrictions. The data that support the findings of this study are available on reasonable request from the corresponding author following the completion of a privacy and fair use agreement.

## Appendix A. 64 Items in the Initial Version of the Six-Facet Artificial Intelligence Literacy Questionnaire (SFAILQ)

1. I consider AI products to be particularly intriguing.
2. I am very curious about how to use new AI products.
3. I have a strong appreciation for the use of AI products.
4. The integration of AI into my life has significantly enriched my sense of enjoyment.
5. I am very interested in exploring emerging AI technologies.
6. I am deeply concerned that AI products may displace a substantial number of jobs.
7. I am concerned that using AI could impair my clear and independent thinking.
8. I find AI products to be somewhat unwieldy in terms of usability.
9. I am highly reluctant to engage with or utilize AI products.
10. I am confident in my ability to address the challenges associated with using AI.
11. I am confident in my capacity to rapidly acquire knowledge about AI.
12. I am confident in my ability to swiftly acquire skills for utilizing AI.
13. I am confident in my ability to effectively leverage AI for a range of tasks.
14. I am confident in my ability to effectively utilize AI to address everyday challenges.
15. I am proficient in using AI products to assist with my learning or work.
16. I often find it challenging to learn how to use a new AI product.
17. I can leverage AI products to improve my learning or work efficiency.
18. I can evaluate the strengths and weaknesses of an AI product following a period of use.
19. I can choose an appropriate option from the diverse solutions offered by the AI application.
20. I can identify the most appropriate AI product from a variety of specific tasks.
21. I can creatively integrate information on the basis of feedback from AI.
22. Using AI often helps me creatively address challenges in my learning or work.
23. I can create a more innovative solution by utilizing the feedback provided by AI.
24. I can arrive at a more viable solution by incorporating the feedback provided by AI.
25. In learning or at work, I can effectively manage the time I allocate to utilizing AI.
26. I can control my frequency of using AI in learning or at work.
27. In learning or at work, I do not rely completely on AI to arrive at an answer.
28. In learning or at work, I do not rely completely on AI to complete tasks on my behalf.
29. In learning or at work, I cannot tolerate not being able to use AI tools.
30. The inability to utilize AI while studying or working has a considerable effect on my mood.
31. In learning or at work, I have not reduced my input due to the use of AI.
32. I have not seen a decline in my academic performance or work efficiency from using AI.
33. I can distinguish between AI devices and non-AI devices.
34. I am uncertain about how AI technology can assist me.
35. I can identify which AI technologies are present in the products I use.
36. I can see the significant impact that AI technology will have on society’s development.
37. I can feel the immense impact that AI has on people’s daily lives.
38. I understand the limitations of AI in handling some complex tasks.
39. I am aware that AI can potentially generate information that is factually inaccurate.
40. I am aware that AI-generated content may exhibit bias and unfairness.
41. For different tasks, I know how to choose and use different AI tools.
42. I am aware of the areas where AI can enhance my learning or work efficiency.
43. When faced with a problem, I am fully aware of whether it is necessary to use AI.
44. I am aware that overreliance on AI may bring about some negative impacts.
45. I stay updated with the latest AI knowledge.
46. I keep track of the latest AI technologies in a timely manner.
47. I continuously improve my skills in using AI.
48. I proactively use AI to increase my learning or work efficiency.
49. I actively utilize AI to address challenges in my daily life.
50. I make comprehensive use of AI to enhance my quality of life.
51. I am becoming more skilled at mitigating the negative impacts of AI.
52. I actively utilize AI to assist my family in solving problems.
53. I actively employ AI to assist my friends in resolving their problems.
54. I aspire to utilize AI to increase the quality of life for my family and friends.
55. When using AI products, I always abide by ethical norms
56. When using AI products, I am always vigilant about privacy and information security issues.
57. I am particularly vigilant against the abuse of AI technology
58. I believe that ethical issues should receive special attention during the development of AI.
59. I assert that the use of AI products must conform to ethical standards and legal regulations
60. I evaluate whether AI responses comply with ethical and legal standards.
61. If I accept the advice of AI, then it means that I should be responsible for it.
62. I am convinced that no one can fully relinquish their personal responsibilities to AI
63. I fully understand that no one should employ AI to inflict harm on others.
64. I contend AI products’ design should focus on preventing potential harm to others

## Appendix B. Six-Facet Artificial Intelligence Literacy Questionnaire (SFAILQ)

### Appendix B.1. Affective Experiences

AE01, I consider AI products to be particularly intriguing.

AE02, I am very curious about how to use new AI products.

AE03, I have a strong appreciation for the use of AI products.

AE04, The integration of AI into my life has significantly enriched my sense of enjoyment.

AE05, I am very interested in exploring emerging AI technologies.

### Appendix B.2. Usage Skills

US01, I am confident in my ability to swiftly acquire skills for utilizing AI.

US02, I am confident in my ability to effectively leverage AI for a range of tasks.

US03, I am confident in my ability to effectively utilize AI to address everyday challenges.

US04, I am proficient in using AI products to assist with my learning or work.

US05, I can evaluate the strengths and weaknesses of an AI product following a period of use.

### Appendix B.3. Cognitive Evaluation

CE01, I can distinguish between AI devices and non-AI devices.

CE02, I can identify which AI technologies are present in the products I use.

CE03, I can see the significant impact that AI technology will have on society’s development.

CE04, I understand the limitations of AI in handling some complex tasks.

CE05, I am aware that AI can potentially generate information that is factually inaccurate.

CE06, When faced with a problem, I am fully aware of whether it is necessary to use AI.

### Appendix B.4. Ethical Norms

EN01, I believe that ethical issues should receive special attention during the development of AI.

EN02, I assert that the use of AI products must conform to ethical standards and legal regulations.

EN03, I evaluate whether AI responses comply with ethical and legal standards.

EN04, I am convinced that no one can fully relinquish their personal responsibilities to AI.

EN05, I fully understand that no one should employ AI to inflict harm on others.

EN06, I contend AI products’ design should focus on preventing potential harm to others.

### Appendix B.5. Responsible Use

RU01, In learning or at work, I can effectively manage the time I allocate to utilizing AI.

RU02, I can control my frequency of using AI in learning or at work.

RU03, In learning or at work, I do not rely completely on AI to arrive at an answer.

RU04, In learning or at work, I do not rely completely on AI to complete tasks on my behalf.

### Appendix B.6. Self-Development

SD01, I stay updated with the latest AI knowledge.

SD02, I keep track of the latest AI technologies in a timely manner.

SD03, I continuously improve my skills in using AI.

SD04, I proactively use AI to increase my learning or work efficiency.

SD05, I actively utilize AI to address challenges in my daily life.

SD06, I make comprehensive use of AI to enhance my quality of life.
